# Evaluating Patient Safety Competency Acquisition Through Clinical Simulation: Development and Application of the Clinical Simulation Learning Patient Safety Scale

**DOI:** 10.1155/nrp/7615766

**Published:** 2026-05-29

**Authors:** Estel·la Ramírez-Baraldes, Daniel García-Gutiérrez, Manuel Romero-Saldaña, Beatriz Rodríguez Diez-Caballero, Rafael González-Moret, Miguel Picher-Martínez, Cristina García-Salido

**Affiliations:** ^1^ Department of Nursing, Faculty of Health Sciences at Manresa, Universitat de Vic-Universitat Central de Catalunya (UVic-UCC), Av. Universitària 4-6, Manresa, 08242, Spain, uvic.es; ^2^ Research Grup in Investigación en Simulación e Innovación Transformativa (GRIST), Instituto de Investigación e Innovación en Ciencias de la Vida y de la Salud de la Cataluña Central (Iris-CC), Ctra. De Roda Núm. 70, Vic, 08500, Spain; ^3^ Intensive Care Unit, Althaia, Xarxa Assistencial Universitària de Manresa, Fundació Privada, Barcelona, Spain; ^4^ GA-16 Lifestyles, Innovation and Health, Maimonides Institute of Biomedical Research of Cordoba (IMIBIC), Córdoba, Spain; ^5^ Department of Nursing, Pharmacology and Physiotherapy, University of Cordoba, Córdoba, Spain, uco.es; ^6^ Department of Nursing, Faculty of Health Sciences at Alfara, Universidad Cardenal Herrera-CEU, CEU Universities C/ Luis Vives s/n, Alfara del Patriarca, 46115, Spain; ^7^ Faculty of Health Sciences, Pre-Departmental Nursing Unit, Universitat Jaume I, Castellón, Spain, uji.es

**Keywords:** clinical simulation, nursing, patient safety, scale validation, transversal competencies

## Abstract

**Introduction:**

Simulation is recognized as an effective method for integrating theory and practice, improving safety competence in nursing professionals and patient care.

**Objectives:**

To develop and psychometrically validate the Clinical Simulation Learning Patient Safety Scale (CSL Patient Safety Scale), a self‐administered instrument for undergraduate nursing students that assesses patient safety competencies acquired through clinical simulation.

**Methods:**

A multicenter study was designed in two Spanish universities, with 450 students and a final sample of 208.

**Results:**

The questionnaire showed a high content validity (Aiken’s V > 0.8) and internal reliability (Cronbach’s *α* = 0.832) with acceptable reliability for each factor separately (*α* = 0.877 for Factor 1 and *α* = 0.714 for Factor 2) and did not present significant variations in sensitivity analysis (McDonald’s *ω* = 0.812). Intraobserver reliability was high (ICC = 0.959, 95% CI = 0.922–0.978).

**Conclusions:**

The questionnaire demonstrated excellent validity and reliability, consolidating the Patient Safety Scale (CSL) as an effective tool for assessing patient safety competencies through clinical simulation.

**Summary**
• Implications for the profession and/or patient care: This scale will provide information to assess student knowledge obtained through clinical simulation.
• Impact**:** It is a tool for the evaluation of transversal competencies, with the improvement of the training of students and future nurses.
• Reporting method: The instructions of the STROBE checklist have been followed.
• Patient or public contribution: The students have participated in the knowledge transfer self‐assessment.

## 1. Introduction

The objective of nursing education in Spain is to equip professionals with the necessary capabilities to provide high‐quality and competent care [1]. Competence is described as “the intersection between knowledge, skills, attitudes and values, as well as the mobilization of these components, to transfer them to the context or real situation, creating the best action/solution, to respond to the different situations and problems that arise at any given time, with the resources available” [2]. Consequently, competence‐oriented education requires strategies that support both its cultivation and its evaluation, this latter aspect being essential in defining professional performance, abilities, and practice settings [2]. Within this framework, patient safety competency refers to the integrated knowledge, skills, and attitudes that enable nursing students to prevent harm and deliver safe care, consistent with patient safety as the “set of structural elements, processes, instruments and methodologies…which aim to minimize the risk of suffering an adverse event in the health care process or to mitigate its consequences” [3].

Accurate competence evaluation depends on the use of tools that meet the standards of validity and reliability. Validity refers to how well an instrument measures what it is intended to measure and whether it serves its original purpose. Reliability, on the other hand, reflects the accuracy and consistency of an instrument when applied under similar conditions. It involves factors such as internal consistency, temporal stability, and inter‐rater reliability [4]. Inter‐rater reliability assesses the level of agreement between two or more evaluators, measuring whether consistent results are obtained when the same scenario is assessed under the same conditions by different individuals [5].

Ensuring patient safety is a fundamental aspect of competence, crucial for delivering care that is both effective and free of harm. Therefore, enhancing care quality has become a central goal across health systems, emerging as both a necessity and a strategic priority in healthcare policies. Patient safety is defined as “the set of structural elements, processes, instruments and methodologies based on scientifically proven evidence, which aim to minimize the risk of suffering an adverse event in the health care process or to mitigate its consequences” [3]. Clinical simulation has proven to be a practical and effective teaching method for fostering a culture of safety in nursing education, thanks to its integration of theory and practice [6].

Viewing safety competence from a comprehensive perspective allows for the inclusion of general attributes and highlights the relevance of context, clinical judgment, and reflective practice. It acknowledges that competence can be demonstrated in various ways [7, 8]. Within this framework, competence is shaped by specific situations and how the nurse responds to them [9]. As noted by Cantrell [10], having skills without the appropriate rationale can lead to unsafe practice—just as possessing knowledge without the skills to apply it effectively can result in poor outcomes.

Patient safety, recognized as a transversal competency in health education, must be comprehensively acquired to ensure safe care practices. Failing to do so can have significant consequences for patient health and strain healthcare systems due to malpractice. Additionally, it requires the integration of new clinical protocols, diagnostic and therapeutic technologies, and a multidisciplinary perspective [11].

Clinical simulation provides a pedagogical framework in which realistic professional scenarios are recreated at different complexity levels. These scenarios allow students to assume specific roles and make decisions, helping them to build competencies and learn through experiential reflection and error [12]. In recent years, the adoption of simulation has expanded significantly in undergraduate and postgraduate health education, particularly in nursing programs, as research shows it is more effective than traditional instruction [13]. Simulation‐based learning not only shortens students’ learning curves and improves the bridge between theory and practice, but also helps identify learning gaps, enhances patient safety, strengthens curriculum design and content, and supports real‐time clinical decision‐making [14–16].

According to the Health Simulation Dictionary [17], simulation‐based education helps learners build and refine both technical and nontechnical skills. These competencies include communication, leadership, teamwork, situational awareness, decision‐making, resource management, safe clinical practice, event prevention and mitigation, and professionalism. Reflective learning plays a key role in this process by fostering critical thinking in a risk‐free and controlled environment, ensuring safety for both students and hypothetical patients.

Nursing practice demands the acquisition of distinct competencies to guarantee care that is both safe and of high quality. In this setting, clinical simulation serves as a crucial resource for developing and evaluating the technical and nontechnical abilities that nursing students need to master in a safe environment [18]. Although tools already exist to measure nursing competencies, the integration of simulation as a method to promote patient safety competencies remains under‐researched. This study seeks to address this gap by validating a specific tool aimed at assessing how simulation impacts the development of patient safety skills, thereby supporting advancements in hands‐on nursing education [19].

On the one hand, the shift in educational paradigms over the past 2 decades has highlighted the need to improve feedback mechanisms in teaching and learning processes. The creation of instruments that enable such mechanisms [20], or that help assess knowledge transfer from simulation to real‐life practice, is therefore highly relevant [21]. On the other hand, nursing care lacks a substantial body of literature concerning standardized practices, unlike other disciplines where such standardization is well‐established [22]. From a nursing perspective, standardizing care implies the pursuit of patient‐centered clinical practice models based on evidence and designed to structure care processes. Applying standardization within simulation scenarios can thus help reduce errors and risks in clinical settings [23].

The CSL Patient Safety Scale was conceived as a self‐report instrument that captures students’ perceived demonstration of patient safety during clinical simulation and the perceived transfer of these competencies to clinical practice. From a KSA perspective, the scale encompasses knowledge, skills and attitudes related to patient safety, operationalized through observable behaviors such as correct patient identification, maintenance of a safe environment, hand hygiene and allergy verification, as well as perceived impact of simulation on knowledge, communication, and technical skills. In relation to Kirkpatrick’s model, the instrument primarily targets the reaction and learning levels, as it assesses students’ self‐perceived acquisition and application of patient safety competencies, rather than objective performance or patient or system‐level outcomes.

Although several instruments such as the Creighton Simulation Evaluation Instrument (C‐SEI) and the Simulation Effectiveness Tool—Modified (SET‐M) provide robust measures of simulation‐related outcomes, they primarily focus on general clinical competence or perceived learning and satisfaction rather than on the specific transfer of patient safety competencies from simulation to real clinical practice. Moreover, there is a lack of validated tools that conceptualize patient safety as a transversal competency and explicitly assess safety‐related behaviors and perceived knowledge transfer in undergraduate nursing curricula. This gap in the existing measurement tools underpins the need to develop a dedicated scale focused on patient safety competence within the context of clinical simulation.

Therefore, the aim of this study was to develop and validate the Clinical Simulation Learning Patient Safety Scale (CSL Patient Safety Scale), a self‐report questionnaire designed for undergraduate nursing students to assess patient safety competencies fostered by clinical simulation, including behaviors related to safe patient identification, environment and procedure, and the perceived transfer of these competencies to clinical practice.

## 2. Methods

### 2.1. Study Design

This was a multicenter, observational study with an instrumental design focused on the development and psychometric validation of the CSL Patient Safety Scale in undergraduate nursing students from two Spanish universities: the Fundación Universitaria del Bages (Manresa Campus) of the UVic‐UCC University and the CEU Cardenal Herrera University (Valencia, Alicante and Castellón campuses).

Both institutions are private centers in Spain that invest in technology and innovative teaching methods, such as clinical simulation in the case of health programs, to improve the learning process and offer practical, up‐to‐date training. Their populations were very similar in terms of general characteristics and capacity.

### 2.2. Creation of the Scale

The development of the CSL Patient Safety Scale followed three main stages: item generation and expert review, content validation and pilot testing, and psychometric validation with a larger student sample.

**FIGURE 1:**
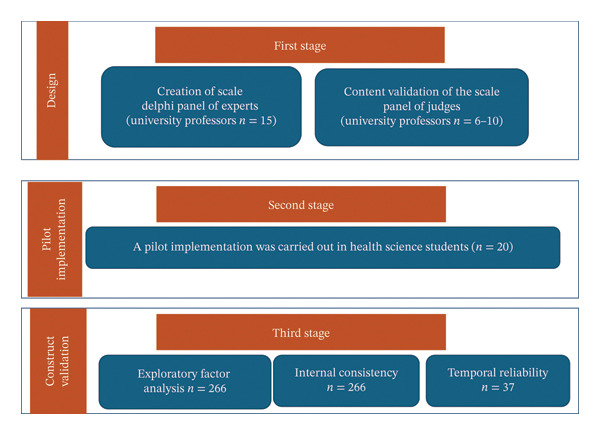
Different stages of design and validation of the *CSL Patient Safety Scale*. Source: own elaboration (2024).

Three distinct stages were carried out (Figure 1). The first stage focused on the development of the instrument, during which the different dimensions were defined based on a review of the literature. Construct dimensions refer to the theoretical domains of patient safety competencies (e.g., knowledge application in safe behaviors and perceived learning outcomes from simulation), derived from literature review and expert input. Based on these dimensions and the characteristics of clinical simulation, the items of the scale were generated with the collaboration of 15 experts using the Delphi method, followed by content validation through the assessment of clarity, importance and relevance by 6–10 judges. The second phase consisted of a pilot test with undergraduate nursing students (*n* = 20) to ensure that understanding of the different items was optimal. In the last phase, psychometric validation took place through factor analysis, internal consistency (*n* = 266), and reproducibility or stability (repeatability) in response after 15 days (*n* = 37).

### 2.3. Procedure

An instrument was developed based on patient safety competence in the Nursing Degree at national level, by means of a literature review. A group of experts made up of 15 specialists was divided into five areas of expertise (three experts per area): clinical simulation, competencies/education, scale validation, healthcare personnel, and Spanish‐language experts. A panel of six judges to ensure the quality and understanding of the content. Consensus was reached through the Delphi method [24].

The 15 experts included nursing educators, clinical simulation specialists, competency researchers, scale validation experts, and healthcare professionals, all fluent in Spanish. The six judges were lecturers from Nursing Degrees with ≥ 15 years’ experience in clinical simulation.

### 2.4. Building the Content

The first part was designed to obtain information from the participants, while maintaining maximum confidentiality. The second part focused on reaching a consensus on a series of items based on patient safety, with the aim of finding out whether what the student applies during the internship was acquired, according to their own perception, through clinical simulation using a Likert‐type response scale with four options: never, some of the time, quite often, or always. This phase was designed with the objective, target population, format, length, and response time in mind. ^1^Its creation was carried out by teachers of the Degree in Nursing, based on competencies that the student must acquire during their studies and experts in clinical simulation using the Delphi method [24] understood as “a method of structuring a group communication process that is effective in allowing a group of individuals, as a whole, to deal with a complex problem.” The aim of this technique is to obtain the degree of consensus or agreement of the specialists on the problem posed, using the results of previous research, rather than leaving the decision to a single professional. [25]. The process ended when saturation was reached in the contributions to the structure of the items and interpretation of the scale, achieving a consensus of over 95% among the experts [26, 27].

### 2.5. Content Validation and Pilot Study

Once the first version of the questionnaire (28 items) had been obtained, content validation began by means of a new Delphi method. For this occasion, a total of six judges were selected, composed of lecturers from the Nursing Degree, with extensive experience in clinical simulation, who evaluated, for each item of the questionnaire, the attributes of “clarity,” “importance,” and “relevance,” by using a Likert‐type response scale of five options, where 1 (worst possible result) to 5 (best possible result). In addition, judges were asked to suggest modifications to the wording of the items to improve the clarity or intelligibility of the items.

The process ended when, after making the appropriate modifications based on the suggestions made, saturation was reached in the judges’ contributions. Next, a pilot test was carried out with 20 students of the Degree in Nursing, using the questionnaire, with the aim of exploring the difficulties they encountered when completing it: ambiguous questions or comprehension difficulties; and duration and type of format, which on this occasion was electronic. After collecting all the impressions, the final version of the questionnaire (nine items) was drafted [28, 29].

The pilot test involved 20 undergraduate nursing students (convenience sample from second–fourth years) who completed the questionnaire and provided qualitative feedback via open‐ended questions on item clarity, comprehension difficulties, completion time (2–5 min), and format preferences. No major issues were identified; minor wording adjustments were made based on feedback. Examples of retained items include “When you have presented yourself to the user, have you acted based on what you have learned in clinical simulations?” (skill transfer); “Do you consider that clinical simulation has helped you to improve technical skills?” (perceived learning).

### 2.6. Population Sample

The estimated total population of undergraduate nursing students at the selected institutions was 450 among all participating universities (Fundación Universitaria del Bages [Manresa Campus] of the UVic‐UCC University and the CEU Cardenal Herrera University [Valencia, Alicante, and Castellón campuses]) and in an equitable manner. A convenience sampling method was used to recruit students enrolled in the bachelor’s degree in nursing program who were in their second to fourth years during the 2023–2024 academic year. The target sample size was determined to be 200 participants, based on anticipated communalities ranging from 0.4 to 0.7 and a low number of variables of 3–4 per factor [26]. The final sample consisted of 208 students, with a response rate of 0.46 (46%).

The students were contacted by email, with voluntary participation and without financial or other compensation. Inclusion criteria included students enrolled in the second, third, or fourth year of the Bachelor of Nursing degree at one of the selected universities, who participated in clinical simulations and clinical practice during the 2023–2024 academic year. Exclusion criteria included students in the first year of the Bachelor of Nursing degree and exchange, validation, or transfer students between universities not included in the study. The questionnaire was administered online and was common to all participating universities and was conducted using the RedCap platform and hosted on the servers of the Fundación Universitaria del Bages (Manresa Campus) of the UVic‐UCC University. All participants completed the questionnaire and were identified by a unique code consisting of the first two letters of their first name, the first two letters of their first surname, the first two letters of their middle name, and their year of birth. In addition, the identification of the center/university from which they were participating was also collected. This last point helped to ensure that our survey was equitable for all centers. To collect all the necessary information, two rounds of reminders were sent to all participants (via email) and in parallel to all participating centers.

Among invited students, nonparticipation was presumed to be mainly related to absence on the day of data collection or lack of time/interest, as students could simply decline to participate or not sign the consent form in this voluntary study. For the test–retest subsample, attrition was mostly explained by timetable incompatibilities and clinical placements 15 days after initial administration.

**FIGURE 2:**
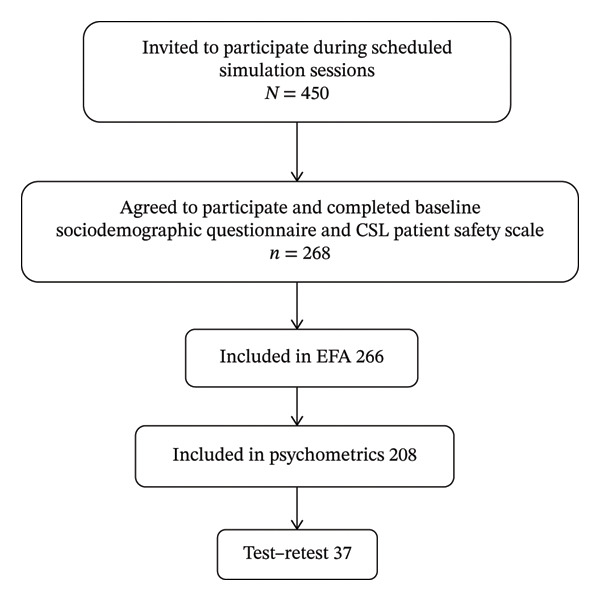
Flow of undergraduate nursing students through eligibility, participation, and analyses. Source: own elaboration (2024).

The methods for evaluating the information were the same for all centers and had been agreed upon in advance. This standardization of methods facilitated the comparison and harmonization of results between the different participating centers. During the academic year 2023–2024, all undergraduate nursing students enrolled in the second, third, or fourth year at the four participating campuses who met the inclusion criteria were considered eligible (*N* = 450). Of these, the students were invited to take part in the study during their scheduled simulation sessions throughout the 2023–2024 academic year, and 450 agreed to participate. A total of 268 students completed the sociodemographic questionnaire and the CSL Patient Safety Scale at baseline and were included in the primary construct validity analyses, and 208 were included in the psychometric analyses for the test–retest subsample, and 37 students from this baseline sample were recontacted 15 days later and [retest_completed] completed the second administration. The flow of participants through the study and the number included in each analysis are summarized in Figure 2.

After data cleaning (exclusion of incomplete cases), final samples were *n* = 266 for exploration factor analysis (EFA), included in reliability analyses (internal consistency) *n* = 208; *n* = 37 for test–retest reliability.

### 2.7. Key Variables and Operational Definitions

The primary outcome variable was patient safety competence in the context of clinical simulation, operationalized through the CSL Patient Safety Scale. For each student, total score (sum of the 9 items divided by 9) and two factor scores correspond to the dimensions identified in the EFA. Each item uses a Likert‐type response format in which higher scores indicate greater perceived demonstration of patient safety behaviors during CLS activities. In addition, several sociodemographic and academic variables were collected as potential predictors and confounders: age (years, continuous), self‐identified gender (female, male, nonbinary), university (UVic‐UCC vs CEU Cardenal Herrera), academic year (second, third, or fourth), and current employment as a health worker (yes/no). Age, gender, and previous work as a health worker were considered a priori as potential confounders or effect modifiers because they may be related both to exposure to patient safety content and to the development of patient safety competencies; therefore, they are reported in detail in Table 1 and were considered when interpreting the generalizability of the findings. No additional effect modifiers were prespecified, and no multivariable models were fitted, as the main objective of the study was to examine the psychometric properties of the CSL Patient Safety Scale rather than to model associations between predictors and outcomes.

**TABLE 1:** Sociodemographic and academic characteristics of participants included in the psychometric validation.

Variable	Total *n* = 266 Mean (SD) *n* (%)	Women *n* = 220 Mean (SD) *n* (%)	Men *n* = 43	Nonbinary *n* = 3	*p*
**AGE**	24.6 (5.8)	24.3 (5.9)	26 (5.4)	23.5 (3.9)	0.21
**UNIVERSITY**					
UVic‐UCC	149 (56%)	124 (83.2%)	22 (14.8%)	3 (2%)	0.14
CEU	117 (44%)	96 (82.1%)	21 (17.9%)	—	
**COURSE**					
SECOND	91 (34.2%)	81 (89%)	8 (8.8%)	2 (2.2%)	0.11
THIRD	19 (7.1%)	14 (73.7%)	5 (26.3%)	—	
FOURTH	156 (58.6%)	125 (80.1%)	30 (19.2%)	1 (0.6%)	
**HEALTH WORKER**					
YES	132 (49.6%)	100 (75.8%)	30 (22.7%)	2 (1.5%)	0.03

*Note:* The words in bold are the variables that were examined/collected: participants’ age (AGE), participating university (UNIVERSITY), participants’ course (COURSE), and whether they participated with healthcare professionals (Health worker yes).

The primary outcome variable was patient safety competence in the context of clinical simulation, operationalized through the self‐administered CSL Patient Safety Scale.

### 2.8. Addressing Potential Sources of Bias

Several strategies were implemented to minimize potential sources of bias. To reduce selection bias, all undergraduate nursing students in the second, third, and fourth years who met the inclusion and exclusion criteria at the four participating campuses during the 2023–2024 academic year were consecutively invited to participate during their scheduled simulation activities, regardless of academic performance or previous professional experience. To limit measurement bias, the CSL Patient Safety Scale was developed through a multistage process including expert review (Delphi method), pilot testing with students, and formal content validation, and the final questionnaire was self‐administered electronically using identical instructions and format across all sites, without collecting identifying information. Potential recall and social desirability biases related to self‐reported data were addressed by asking students to rate their current simulation‐based learning experiences within the same academic year and by emphasizing that there were no right or wrong answers and that their responses would be anonymous and would not affect their academic evaluation.

### 2.9. Psychometric Properties

Reliability or internal consistency, intraobserver reliability (temporal stability) and an exploratory factor analysis, reliability or internal consistency, and intraobserver reliability (temporal stability) were analyzed to determine the psychometric characteristics of the scale.

### 2.10. Statistical Analysis

For content validation, the Aiken V‐statistic was used, considering cutoff points of values above 0.8 for all items [27].

#### 2.10.1. Exploratory Factor Analysis (EFA)

For psychometric validation, an EFA was conducted using the maximum likelihood extraction method and direct Oblimin rotation (Kaiser rotation). In addition, the Kaiser–Meyer–Olkin (KMO) test was used to estimate the sample adequacy measure, Bartlett’s test of sphericity to test the null hypothesis of the correlation matrix, the value of the determinant of the correlation matrix to check whether the variables of the questionnaire were linearly related, and the sedimentation plot for the visualization of the extracted factors.

#### 2.10.2. Internal Consistency

The internal consistency or reliability was assessed using Cronbach’s alpha and MacDonald’s *ω* coefficient, considering, as reference criteria, values higher than 0.70 [30, 31]. It was considered that it was only necessary to apply Cronbach’s alpha sensitivity analysis, as this already provided valid values. It was considered that performing other sensitivity analyses would not provide a better analysis and that the results would not vary.

#### 2.10.3. Test–Retest Reliability (ICC)

For temporal stability (test–retest or intraobserver reliability), the intraclass correlation coefficient (ICC) and the Bland and Altman plots were determined. The test–retest was performed at an interval of 15 days[32]. As the questionnaire was self‐administered, it was not necessary to assess intraobserver reliability.

Contrast tests were performed with an alpha error of less than 5%, and confidence intervals were calculated with 95% confidence. SPSS Ver. 26 statistical software was used for data analysis.

Before performing the analyses, we examined the pattern of missing data for all variables of interest. No missing data were observed for the sociodemographic variables or for the CSL Patient Safety Scale items, so all available questionnaires were included in the analyses. Although the scale could have subgroups and demographic data collection such as the university of origin, year and course of participants, or work experience, there was no subgroup analysis because the objective was to evaluate the scale and its validation. The results were not being evaluated or compared according to center, year of study, or type of work. Data related to the participant’s university, year of study, or work sector were only used for control purposes to ensure fairness in data collection across all centers.

In the case of surveys with missing data, these were discarded. Before beginning the analysis of the data obtained, all data were cleaned, leaving only those surveys that were 100% complete. A convenience sampling strategy was used as described above, with a multicenter design. Despite this, the data were collected and centralized using a single questionnaire completed with RedCap software centralized for all participating centers. This method subsequently facilitated data analysis. As the study did not intend to compare the data obtained between universities, but rather to validate the scale, it was not necessary to perform a comparative analysis between universities, but rather a unified and common analysis for all of them.

### 2.11. Ethical and Legal Aspects

The research study complied with the principles of the Declaration of Helsinki on Biomedical Research. Furthermore, it obtained authorization from each of the ethics committees of the two institutions participating in the study: the UVic‐UCC Research Ethics Committee (document 141/2001) and the Biomedical Research Ethics Committee of the Cardenal Herrera University (CEU) (document CEE123/442).

All participants were informed of the objectives of the research and agreed to participate in the study after giving their informed consent. The decision not to ask participants about their nonparticipation was considered appropriate, as it was voluntary study, and in the event of not wanting to participate, it was not necessary to communicate the reason, simply stating that they did not want to participate and not signing the consent form was sufficient.

## 3. Results and Discussion

The results are presented following the main stages of the instrument development process: content validation, construct validity, and reliability analyses. Following expert consensus and content validation, the final 9‐item questionnaire was administered to 268 students. Psychometric analyses were then conducted on cleaned data (*n* = 266 for EFA)

### 3.1. Creation of the Scale and Content Validation

Initially, the scale contained 28 items; after three rounds of the Delphi method with the experts, nine items were obtained in the final scale. Once the questionnaire had been created, a group of six judges, made up of lecturers from the Degree in Nursing with at least 15 years’ experience in teaching clinical skills using simulation techniques, participated in the content validation of the questionnaire by assessing the “clarity,” “importance,” and “relevance” of each item on a Likert scale of 1–5 points, with 1 being the lowest value and 5 the maximum for each attribute assessed. The results of the Aiken V‐statistic [33] for each item and for the overall scale can be seen in Table 2. The Aiken’s V value for the overall scale was 0.9 CI 95% (0.76–0.96).

**TABLE 2:** Content validation of the scale.

	Item	Clarity	Importance	Relevance
1	SIMULATION: When you have presented yourself to the user, have you acted based on what you have learned in the clinical simulations?	0.833	0.958	0.958
2	SIMULATION: Have you actively identified the users based on what you have learned in the clinical simulations?	0.75	0.958	0.958
3	SIMULATION: When you have approached the buzzer and placed the guardrails on the user, have you done so considering what you have learnt in the simulations carried out?	0.958	0.917	0.917
4	SIMULATION: When you performed the hygienic hand washing and put on the non‐sterile gloves, did you do it based on what you learnt in the simulation?	0.875	0.958	0.958
5	SIMULATION: When you asked about the existence of any type of allergy, did you do it based on what you learned in the simulation?	0.917	0.958	0.958
11	By what percentage do you consider that the simulations carried out during this course are close to the reality of care?	0.875	0.792	0.792
12	Do you consider that the clinical simulation has helped you to increase your knowledge?	0.958	0.917	0.917
13	Do you consider that clinical simulation has helped you to improve your communication skills?	0.917	0.958	0.917
14	Do you consider that clinical simulation has helped you to improve technical skills?	0.958	0.917	0.917
	Overall	**0.921**	**0.929**	**0.917**

*Note:* AIken’s *V* values.

For the content validation, cutoff points were considered values above 0.8 for each of the three attributes of the questionnaire: clarity, importance, and relevance [34–36].

Within this first part of content validity, the group of students in the pilot group, chosen at random, expressed that the questions were easy to understand; their duration was adequate, between 2 and 5 min; the electronic format seemed adequate to them, due to its good visibility and ease of use, so it was considered that the survey was ready to be sent massively to the students who were the object of our study. In the scientific evidence, there are many articles that manage to obtain a validated scale in the field of health and, specifically, in nursing, using the methodology of clinical simulation. Most of them make use of the Delphi method to obtain a consensus or agreement of the specialists on the problem posed, using the results of previous research, instead of leaving the decision to a single professional [25].

Although both instruments, C‐SEI and SET‐M, are well‐established and exhibit strong psychometric properties, their focus and application differ from those of the CSL Patient Safety Scale. The C‐SEI primarily assesses observable student behaviors during simulated clinical scenarios, focusing on clinical judgment and patient safety from the viewpoint of trained external observers. In contrast, the SET‐M captures students’ self‐perceived outcomes of simulation‐based learning, such as confidence, communication, and preparedness for practice. The CSL Patient Safety Scale offers a novel contribution by specifically evaluating the transfer of patient safety competencies, acquired through simulation, to real clinical practice. This focus on transversal competencies within undergraduate nursing curricula fills a gap not fully addressed by the existing tools.

### 3.2. Exploratory Factor Analysis

A total of 266 students were included in the construct validity analyses (EFA) to the Nursing Degree at the Manresa Campus (UVic‐UCC University) and Valencia, Alicante, and Castellón campuses (CEU Cardenal Herrera‐CEU University). The overall mean age was 24.6 (5.8) years 95% CI (23.9–25.3) and range from 19 to 54 years. 82.7% were female (220 students), 156 were fourth‐year undergraduates (58.6%), and 132 had ever worked as healthcare workers (49.6%). Table 1 shows the characteristics of the study sample.

In the application phase of the instrument, 268 surveys were answered, far exceeding the representative sample of our target population. To validate the construction, an EFA was carried out using the maximum likelihood extraction method and the direct Oblimin rotation method (Kaiser rotation).

In addition, the KMO test was used to estimate the sample adequacy measure, Bartlett’s test of sphericity to test the null hypothesis of the correlation matrix, and the value of the determinant of the correlation matrix to check whether the variables of the questionnaire were linearly related, and the sedimentation plot for the visualization of the extracted factors.

**FIGURE 3:**
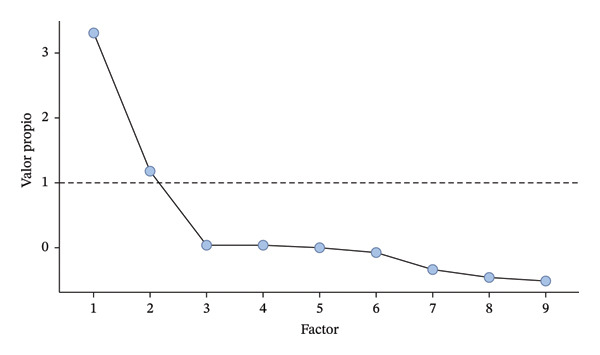
EFA. Sedimentation plot for factor extraction.

The correlation matrix between the nine items of the questionnaire obtained a determinant value of 0.017. Furthermore, the sample adequacy measure analyzed by the KMO statistic reached a result of 0.812 and Bartlett’s test of sphericity was highly significant (*p* < 0.001), thus verifying the suitability of carrying out a PFA. The factor solution proposed two factors or dimensions that explained 53.9% of the total variance of the model. Figure 3 illustrates the sedimentation plot, and Table 3 shows the standard matrix of the rotated solution with factor loadings and uniqueness (all nine items were retained in the final scale; although two items showed lower factor loadings in the EFA, they were preserved based on content validity criteria).

**TABLE 3:** EFA.

	Factor		
	1	2	Uniqueness
Q2_CLINICAL SIMULATION: When you have presented yourself to the user, have you acted based on what you have learned in clinical simulations?		0.666	0.525
Q4_SIMULATION: Did you actively identify users based on what you learned in the clinical simulations?		0.745	0.485
Q6_SIMULATION: When you have approached the buzzer and placed the guardrails on the user, have you done it considering what you have learned in the simulations you have done?		0.626	0.619
Q8_SIMULATION: When you performed the hygienic hand washing and put on the non‐sterile gloves, did you do it based on what you learned in the simulation?		0.476	0.682
Q10_SIMULATION: When you asked about the existence of any type of allergy, did you do it based on what you learned in the simulation?		0.681	0.509
Q11_What percentage do you consider that the simulations carried out during this course are close to the reality of care?	0.602		0.618
Q12_Do you consider that the clinical simulation has helped you to increase your knowledge?	0.918		0.159
Q13_You think that clinical simulation has helped you to improve communication skills	0.776		0.358
Q14_You think that clinical simulation helped you to improve technical skills	0.913		0.191

*Note:* All nine items were retained in the final scale; Item Q8 showed loading < 0.5 but was kept for content validity. Extraction method: maximum likelihood. Rotation method: Oblimin Kaiser. Rotation has converged in three iterations. Pattern matrix of the rotated solution with factor loadings and uniqueness.

It is observed that all factor loadings of the standard matrix obtained values above 0.5 except for Item 8. Factor 1 included the items related to the dimension “Skill acquisition,” while **Factor 2** included those items related to the dimension “Knowledge transfer.” Finally, the factor correlation matrix yielded a correlation coefficient of 0.357 between Factors 1 and 2.

The two‐factor solution (Skill acquisition: Items 2, 4, 6, 8, 10; loadings 0.476–0.745; Knowledge transfer: Items 11–14; loadings 0.602–0.918) explained 53.9% variance (factor correlation *r* = 0.357). This bifactor structure aligns with simulation scales assessing competency transfer, where two dimensions commonly emerge (technical skills + perceived learning), differing from SSES’s three‐factor model (debriefing, clinical reasoning, learning; *n* = 268, *α* = 0.77), which targets broader simulation experience [37]. Our parsimonious structure suits patient safety’s focused assessment needs.

### 3.3. Internal Consistency and Stability

The CSL Patient Safety Scale demonstrated excellent internal consistency across its nine items (2, 4, 6, 8, 10, 11, 12, 13, 14) with Cronbach’s *α* = 0.832 (95% CI: 0.779–0.856) and McDonald’s *ω* = 0.812 (95% CI: 0.794–0.868) in the sample of 208 students; sensitivity analysis confirmed no variation upon item deletion.

Internal consistency was excellent for the total scale (Cronbach’s *α* = 0.832). Separate factor analyses revealed acceptable reliability for the skill acquisition dimension (*α* = 0.877) and the knowledge transfer dimension (*α* = 0.714).

The factor solution identified two dimensions explaining 53.9% of the total variance. Factor 1 (skill acquisition) included items related to perceived learning outcomes from simulation (Q11–Q14; loadings 0.602–0.918), whereas Factor 2 (knowledge transfer) comprised items reflecting the application of patient safety behaviors in clinical simulation (Q2, Q4, Q6, Q8, Q10; loadings 0.476–0.745). All items showed satisfactory factor loadings (> 0.50), except Item Q8 (loading = 0.476), which was slightly below the conventional threshold. However, this item was retained due to its high content validity and its relevance as a core patient safety behavior (hand hygiene). The factor correlation matrix indicated a moderate correlation between both dimensions (*r* = 0.357), supporting the conceptual distinction between skill acquisition and knowledge transfer while confirming their related nature within patient safety competence. This two‐factor structure is consistent with competency‐based frameworks distinguishing between behavioral performance (skills) and perceived learning outcomes (knowledge transfer).

Internal consistency analysis by dimension was additionally performed. Cronbach’s alpha was calculated separately for the skill acquisition dimension (Items Q2, Q4, Q6, Q8, Q10) and the knowledge transfer dimension (Items Q11–Q14), both showing acceptable levels of internal consistency.

**FIGURE 4:**
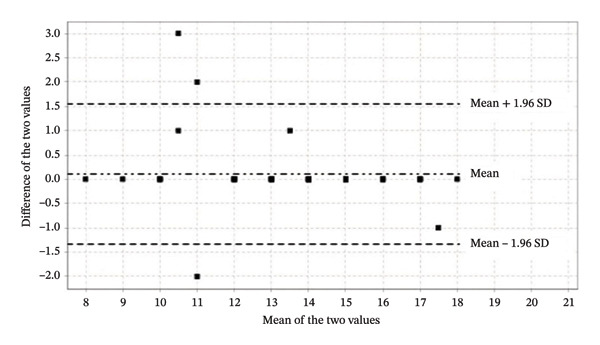
Concordance between test–retest scores. Bland and Altman plot.

Test–retest reliability was equally robust among 37 nursing students (second–fourth years) assessed over a 15‐day interval, yielding ICC = 0.959 (95% CI: 0.922–0.978). Figure 4 (Bland–Altman plot) confirms excellent temporal stability, with differences tightly clustered around the mean (no systematic bias) and within narrow limits of agreement (±1.96 SD), indicating consistent scores across administrations.

These reliability estimates exceed acceptability thresholds (*α*/*ω* > 0.70) and compare favorably to established simulation scales: SET‐M (*α* = 0.93, more items) and observer‐rated C‐SEI (*α* = 0.979), validating CSL for self‐reported patient safety competency assessment [38, 39].

### 3.4. Applicability to Other Nursing Student Populations and Educational Contexts

Although this study was conducted with undergraduate nursing students from two private Spanish universities with strong investment in simulation‐based education, the competencies targeted by the CSL Patient Safety Scale (e.g., patient identification, safe environment management, hand hygiene, allergy verification, and communication) reflect core patient safety behaviors that are expected of novice nurses in most healthcare systems. For this reason, we consider that both the scale and the findings are potentially applicable to other nursing student populations and to programs that use clinical simulation as a key teaching–learning strategy, provided that appropriate linguistic and cultural adaptation is undertaken. In educational contexts with different curricular structures, resource levels, or patient safety cultures, the CSL Patient Safety Scale could be used after additional local validation to monitor the acquisition and transfer of patient safety competencies, and future studies should explore its performance in public universities, in other countries, and in interprofessional or postgraduate training settings.

## 4. Limitations

Among the limitations of this study is that there are no scientific studies that explore and analyze the transfer of competencies from clinical simulation to healthcare practice. This fact, which makes discussion with other authors difficult, gives this research a very positive factor based on its originality.

With reference to the characteristics of our population, it can be observed that the number of women is five times higher than that of men. This proportion reflects the actual gender distribution of the nursing profession in Spain, a female profession in the last 20 years [40]. This gender imbalance requires a study where more male or nonbinary samples are obtained to explore whether there is a difference in the perception of learning linked to gender. There are studies that identify that the degree of satisfaction, by gender in simulation, is different [41].

In addition, because patient safety competencies and the contribution of clinical simulation were assessed using self‐reported data, some residual selection and information biases (including recall and social desirability bias) may persist despite the measures implemented to minimize them.

## 5. Conclusions

The CSL Patient Safety Scale demonstrates robust psychometric properties for assessing patient safety competencies acquired through clinical simulation among undergraduate nursing students. This 9‐item, self‐administered instrument exhibits high content validity (Aiken’s V > 0.8 across clarity, importance, and relevance), excellent internal consistency (Cronbach’s *α* = 0.832; McDonald’s *ω* = 0.812), and strong test–retest reliability (ICC = 0.959), confirming its suitability as a brief, reliable evaluation tool.

Exploratory factor analysis revealed a parsimonious two‐factor structure explaining 53.9% variance: Skill acquisition (patient identification, safe environment management, hygiene protocols, allergy verification) and ^∗^Knowledge transfer^∗^ (perceived simulation realism and learning outcomes). Unlike satisfaction‐focused scales, CSL specifically measures the transfer of safety competencies from simulation to clinical practice, complementing observer‐rated instruments like C‐SEI (*α* = 0.979) and general learning measures like SET‐M (*α* = 0.93).

Integration of CSL into nursing curricula will enable educators to quantify simulation impact, identify training gaps, and monitor longitudinal competency development, ultimately fostering a culture of safety from undergraduate training and preparing graduates for safe clinical practice.

## Funding

No funding was received for this manuscript.

## Conflicts of Interest

The authors declare no conflicts of interest.

## Endnotes

^1^It involves a combination of practical skills (knowledge, motivation, ethical values, attitudes, emotions, skills, abilities, aptitudes, and other social and behavioral components that are mobilized together to achieve effective action). They are thus seen as knowledge in practice, i.e., knowledge acquired through participation in social practices and, as such, can be developed both in the formal educational context, through the curriculum, and in nonformal and informal educational contexts. Competences can therefore be defined as “know‐how,” i.e., knowledge acquired through participation in social practices.

## Supporting Information

Additional supporting information can be found online in the Supporting Information section.

## Supporting information


**Supporting Information** STROBE statement—checklist of items that should be included in reports of ***cross-sectional studies***.

## Data Availability

The data that support the findings of this study are available from the corresponding author upon reasonable request.
